# Health-related quality of life and associated factors among epilepsy patients in sub-Saharan Africa: a systematic review and meta-analysis

**DOI:** 10.3389/fneur.2025.1546911

**Published:** 2025-03-05

**Authors:** Dessale Abate Beyene, Desalegn Getnet Demsie, Chernet Tafere, Taklo Simeneh Yazie, Destaw Endeshaw, Tamrat Assefa Tadesse, Zenaw Debasu Addisu

**Affiliations:** ^1^Department of Pharmacy, Asrat Woldeyes Health Science Campus, Debre Berhan University, Debre Birhan, Ethiopia; ^2^Department of Pharmacology, College of Medicine and Health Sciences, Bahir Dar University, Bahir Dar, Amhara, Ethiopia; ^3^Department of Pharmaceutics, College of Medicine and Health Sciences, Bahir Dar University, Bahir Dar, Amhara, Ethiopia; ^4^Pharmacology and Toxicology Unit, Department of Pharmacy, College of Health Sciences, Debre Tabor University, Debre Tabor, Ethiopia; ^5^Department of Adult Health Nursing, College of Medicine and Health Sciences, Bair Dar University, Bahir Dar, Amhara, Ethiopia; ^6^Department of Pharmacology and Clinical Pharmacy, School of Pharmacy, College of Health Sciences, Addis Ababa University, Addis Ababa, Ethiopia; ^7^Department of Clinical Pharmacy, College of Medicine and Health Sciences, Bahir University, Bahir Dar, Amhara, Ethiopia

**Keywords:** health-related quality of life, people living with epilepsy, mean score, sub-Saharan Africa, associated factors

## Abstract

**Background:**

Epilepsy is a major public health issue worldwide, often leading to physical and cognitive impairments that limit employment, independence, and social interaction. Health-related quality of life (HRQoL) is a crucial outcome in the treatment of chronic epilepsy as it is linked to reduced independence, treatment challenges, and lower life expectancy. HRQoL serves as an important health indicator for assessing the impact of the disease on daily living activities.

**Objective:**

This study aimed to estimate the mean score of health-related quality of life (HRQoL) and factors associated with lower HRQoL in people living with epilepsy (PLWE) in sub-Saharan African (SSA) countries.

**Methods:**

A comprehensive literature search was conducted using PubMed, Cochrane Library, Scopus, and Google Scholar databases. This review has been registered with PROSPERO (CRD42024620363). The eligibility criteria were established, and this review included cross-sectional and observational studies assessing HRQOL in PLWE in SSA countries, published in English from the inception of databases through November 2024. The pooled HRQoL was reported as the mean score with accompanying 95% confidence intervals. Finally, publication bias was evaluated using a funnel plot and Egger’s regression test.

**Results:**

The pooled mean score of HRQoL among PLWE in SSA was 63.79 (95% CI: 59.75–67.84%). Owing to significant heterogeneity across the studies, a random-effects model was utilized for the meta-analysis (I^2^ = 98.96%, *p* < 0.001). This meta-analysis indicated that anxiety (*β* = −4.762, *p* = 0.0029), depression (*β* = −4.591, *p* < 0.0001), uncontrolled seizures (*β* = −4.321, *p* < 0.0001), and a family history of epilepsy (*β* = −5.093, *p* = 0.0013) had statistically significant negative impacts on HRQoL in PLWE. Despite some asymmetry in the funnel plot, Egger’s test showed no significant publication bias, with a *p*-value of 0.321.

**Conclusion:**

This review found a moderate pooled mean score of HRQoL among PLWE in SSA countries. Factors that negatively affect HRQoL in these regions include anxiety, depression, uncontrolled seizures, comorbidities, and a family history of epilepsy.

**Systematic review registration:**

https://www.crd.york.ac.uk/PROSPERO/search, identifier CRD42024620363.

## Background

Epilepsy is a prevalent chronic neurological disorder characterized by recurrent unprovoked seizures that vary greatly in clinical presentations (1). It is a significant public health concern worldwide and is often linked to physical and cognitive impairments that can restrict employment opportunities, independence, and social engagement (2). Globally, 70 million people suffer from epilepsy, with a high prevalence in developing countries, particularly sub-Saharan African (SSA) countries. Approximately 80% of people living with epilepsy (PLWE) reside in developing countries with limited resources, and as a result, the majority of epilepsy patients remain untreated (3, 4). Epilepsy is a major burden in developing countries, and it affects not only individuals with epilepsy but also their families and society as a whole (5). It is not only a medical problem, but also a social problem that has a negative impact on patients, both socially and culturally (6–8).

Epilepsy has a significant impact on health-related quality of life (HRQoL) (9). The HRQoL of PLWE may be impaired by various factors, including seizure complications, emotional changes (depression and anxiety), stigma, and low social support or social isolation (10). Ineffective Anti-seizure drugs (ASDs) and prolonged disease duration affect the HRQoL of PLWE (11). In SSA, up to 90% of PLWEs have been reported to receive inadequate treatment or no treatment, which significantly affects HRQoL (5). The administration of ASDs to treat seizures and prevent further complications; however, the side effects of these drugs can potentially affect patients’ HRQoL (12).

Epilepsy is a condition that has been culturally devalued worldwide throughout history, and there is often a lack of awareness and understanding of epilepsy, which leads to stigmatization, discrimination, and exclusion of PLWE from society (13). In addition to that epilepsy affects life in many ways and should be studied taking into account the cultural conditions and psychosocial composition of each community (14, 15). PLWE is at risk of physical harm due to the potential dangers of a seizure, which can result in injury or even death (16).

HRQoL is recognized as an important outcome of epilepsy treatment and analysis of HRQoL in patients with chronic epilepsy because it is associated with lower personal independence, treatment-related problems, and lower life expectancy (17). To measure the burden of the disease on daily living activities, HRQOL has been used as a measure of health indicators, apart from morbidity and mortality (18–20). Several instruments have been developed to assess HRQoL in epilepsy patients and frequently used instruments in SSA countries were the World Health Organization’s Quality of Life Questionnaire (WHOQOLBREF), Quality Of Life in Epilepsy (QOLIE)-31, Hospital Anxiety and Depression Scale (HADS) (21–24).

Evaluating the impact of epilepsy on the HRQoL of PLWE in SSA would shed light on the various factors that affect HRQoL (23–27). There is a growing consensus that successful treatment should not only target the severity of symptoms but also impairment of functioning and HRQoL, leading to the restoration of health (22). In SSA countries, there is a lack of reliable evidence for quantifying the impact of epilepsy on HRQoL. The issue of HRQoL assessment and contributing factors is often overlooked in PLWE in SSA countries.

Few studies have been conducted in SSA countries on the HRQoL of PLWE. The reported mean score results showed significant discrepancies, ranging from 49.9% in Uganda (28) to 94.03 ± 10.61% in Nigeria (29). Therefore, it is crucial to estimate the magnitude of this problem and determine the factors that affect the HRQoL of PLWE to develop effective interventions and policy responses that can help improve HRQoL. This study is a comprehensive systematic review and meta-analysis of findings from various SSA countries. The findings of this study can help various organizations working to support PLWE to strengthen their actions in line with the magnitude of the problem.

## Materials and methods

### Protocol and registration

The Preferred Reporting Items for Systematic Reviews and Meta-Analyses (PRISMA) guidelines were used to prepare this systematic review and meta-analysis [SF 1 (PRISMA_2020).docx]. The review protocol is registered with the International Registration of Systematic Reviews (PROSPERO) (30) with, registration number CRD42024620363.

### Study eligibility criteria

This systematic review included all published research on the mean scores of health-related quality of life (HRQoL) and the factors associated with HRQoL in PLWE in Sub-Saharan Africa (SSA). Studies were eligible if they reported HRQoL data using validated instruments and presented the mean HRQoL scores. This review includes cross-sectional, observational, and studies published in English from inception to November 2024.

Conference abstracts, qualitative studies review articles, case–control studies, unpublished works (thesis), commentaries, gray literature, and those not fully accessible were excluded.

### Data source and search strategy

To identify relevant publications for the systematic review and meta-analysis, we conducted both manual and electronic searches. The databases searched included PubMed, MEDLINE, EMBASE, the Cochrane Library, Scopus, and Google Scholar. We employed the following free-text keywords: (“Epilepsy” OR “Seizure Disorder”) AND (“Quality of Life” OR “Health-Related Quality of Life” OR “HRQoL” OR “QoL”) OR (“Associated Factors” OR “Determinants” OR “Predictors”) AND (“Sub-Saharan Africa” OR “Africa South of the Sahara” OR the specific names of Sub-Saharan African countries such as Angola, Benin, Botswana, Burkina Faso, Burundi, Cabo Verde, Cameroon, Central African Republic, Chad, Comoros, Congo, Democratic Republic of Congo, Djibouti, Equatorial Guinea, Eritrea, Eswatini, Ethiopia, Gabon, Gambia, Ghana, Guinea, Guinea-Bissau, Ivory Coast, Kenya, Lesotho, Liberia, Madagascar, Malawi, Mali, Mauritania, Mauritius, Mozambique, Namibia, Niger, Nigeria, Rwanda, Sao Tome and Principe, Senegal, Seychelles, Sierra Leone, Somalia, South Africa, South Sudan, Sudan, Togo, Uganda, Tanzania, Zambia, Zimbabwe).

### Data extraction

Three authors (DAB, ZDA, and DGD) independently extracted the required data from the articles using a format prepared in Microsoft Excel. This form was designed to gather relevant information, including authors’ names, publication years, study country, study designs, settings, participants, sampling methods, sample sizes, study duration in months, mean HRQoL scores with standard deviations, quality of life assessment tools used to assess HRQoL, and factors associated with HRQoL.

### Quality and risk of bias assessment

The Joanna Briggs Institute (JBI) tool for cross-sectional study quality assessment was used to evaluate the methodological quality of the studies included in this review (31). Two authors (ZDA and DGD) independently assessed the quality of the original research using JBI criteria and discrepancies between the two reviewers were resolved through discussion. Studies that scored seven or higher on a nine-point scale were included in the analysis. The JBI tool assesses nine key areas: whether the sample frame is appropriate for addressing the target population if study participants were sampled appropriately, whether the sample size was adequate if study subjects and settings were described in detail, whether data analysis sufficiently covered the identified sample if valid methods were used to identify the condition, whether the condition was measured reliably for all participants, if the statistical analysis was appropriate, and whether an adequate response rate was achieved, or if a low response rate was properly managed.

### Outcome measurement

#### Primary outcome

Health-Related Quality of Life (HRQoL): The main outcome of this study was the pooled estimate of the mean HRQoL scores among PLWE in SSA, which were assessed using validated instruments.

#### Secondary outcome

Associated Factors of HRQoL among PLWE in Sub-Saharan Africa (SSA).

### Data analysis

To estimate the pooled mean HRQoL score, both mean and 95% confidence intervals (CIs) were reported. The mean standard error was calculated by dividing the standard deviation by the square root of the sample size. For the associated factors, odds ratios, logarithms, and standard errors of these logarithms were computed. Data were initially extracted into Microsoft Excel and subsequently imported into STATA 17.0 for further analysis. A random-effects model was employed to summarize the data, while heterogeneity was assessed using the Q test and I^2^ statistic. The thresholds for I^2^, indicating low, moderate, substantial, and high heterogeneity, were defined as ≤25%, 25–50%, 50–75%, and ≥ 75%, respectively. Meta-regression analyses were conducted to assess the impact of continuous and categorical moderator variables (e.g., study year, sample size, and study quality) on HRQoL scores. Additionally, subgroup analyses were performed based on the study region in Africa and the type of HRQoL instrument used to identify potential sources of heterogeneity. Meta-analysis and narrative analyses were used to present the findings. The pooled HRQoL was reported as the mean score with accompanying 95% confidence intervals. A sensitivity analysis was performed to verify that the results were robust against potentially influential decisions. Finally, publication bias was evaluated using a funnel plot and Egger’s regression test. If Egger’s test indicated statistical significance (*p* < 0.05) or if the funnel plot exhibited asymmetry, publication bias was noted.

## Results

### Search results

**Figure 1:**
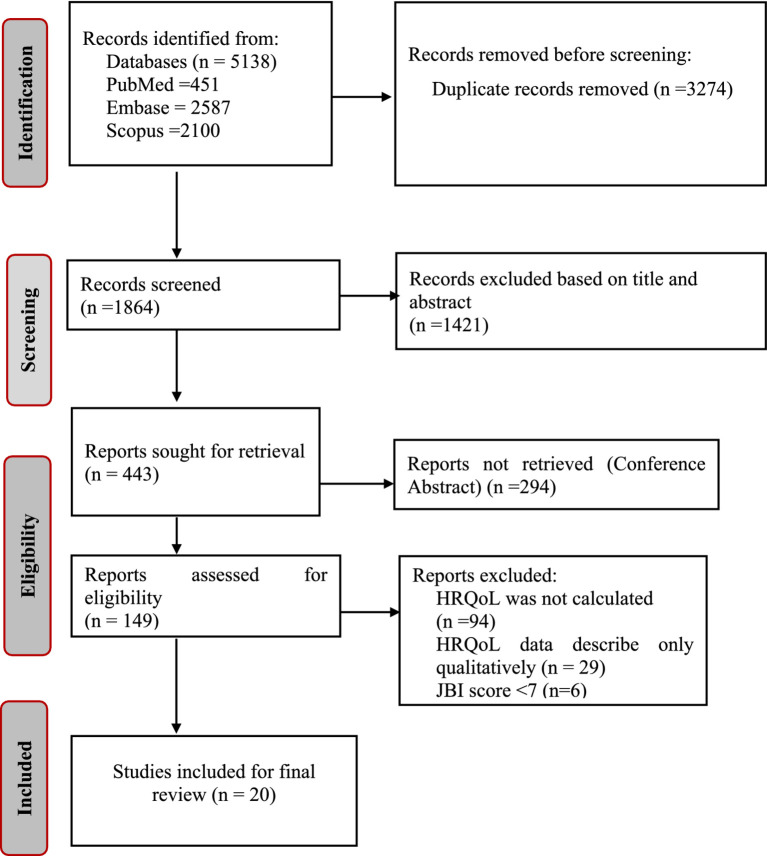
PRISMA Flow diagram for study selection for systematic review and meta-analysis.

A total of 5,138 publications were identified through database searches of PubMed (451), Embase (2,587), and Scopus (2,100). After removing 3,274 duplicates, 1,421 papers were excluded during the title and abstract screening. We attempted to retrieve 443 reports; however, 294 could not be obtained because they were conference abstracts. Of the 149 full-text articles assessed for eligibility, 94 were excluded because HRQoL was not calculated, 29 due to HRQoL data being described only qualitatively, and six due to a JBI score of less than seven. Ultimately, 20 studies were included in this systematic review and meta-analysis (Figure 1).

### Quality and risk of bias assessment

In this meta-analysis, 26 articles were assessed and six articles were excluded because their JBI scores were < seven. A total of 20 studies were included, all of which had a JBI score higher than seven with scores ranging from 77.7 to 100% (Table 1). Only studies that scored seven or higher on a nine-point scale were included in the analysis [SF 2 (JBI).docx].

**Table 1:** Characteristics and quality assessment of studies on the quality of life of people with epilepsy in SSA.

No	Author ID	Study area (Country)	Regions in Africa	Sample size	Study design	Tool	Minimum and maximum score	Mean score HRQoL	JBI-Quality score
1	Mesafint et al. (22)	Ethiopia	East Africa	439	Cross-sectional	WHOQOL-BREF	0 to 100	61.1 ± 11.6	100
2	Tegegne et al. (27)	Ethiopia	East Africa	415	Cross-sectional	WHOQOL-BREF	0 to 100	56.36 ± 13.37	88.9
3	Tefera et al. (21)	Ethiopia	East Africa	121	Cross-sectional	WHOQOL-BREF	0 to 100	56.42 ± 10.96	77.7
4	Kassie et al. (32)	Ethiopia	East Africa	395	Cross-sectional	QOLIE-31	0 to 100	63.43 ± 26.56	100
5	Abadiga et al. (40)	Ethiopia	East Africa	392	Cross-sectional	WHOQOL-BREF	0 to 100	60.47 ± 23.07	88.9
6	Shiferaw et al. (33)	Ethiopia	East Africa	304	Cross-sectional	QOLIE-31	0 to 100	58.8 ± 10.6	77.7
7	Gebre et al. (26)	Ethiopia	East Africa	175	Cross-sectional	QOLIE-31	0 to 100	77.97 ± 20.78	88.9
8	Addis et al. (24)	Ethiopia	East Africa	376	Cross-sectional	QOLIE-31	0 to 100	55.81 ± 14	77.8
9	Adewuya et al. (43)	Nigeria	West Africa	86	Cross-sectional	QOLIE-AD-48	0 to 100	66.8 ± 5.04	77.7
10	Ogundare et al. (34)	Nigeria	West Africa	270	Cross-sectional	QOLIE-31	0 to 100	77.98 ± 13.32	88.9
11	Nabukenya et al. (28)	Uganda	East Africa	175	Cross-sectional	QOLIE-31	0 to 100	58 ± 13	77.7
12	Kaddumukasa et al. (35)	Uganda	East Africa	48	Cross-sectional	QOLIE-31	0 to 100	62.5 ± 14.5	88.9
13	Iwuozo et al. (41)	Nigeria	West Africa	103	Cross-sectional	WHOQOL-BREF	0 to 100	65.2 ± 16.4	77.7
14	Fawale et al. (36)	Nigeria	West Africa	93	Cross-sectional	QOLIE-31	0 to 100	64.2 ± 13.6	88.9
15	Amaral et al. (37)	Tanzania	East Africa	96	cross-sectional	QOLIE-31	0 to 100	66.9 ± 13	88.9
16	Onwuekwe et al. (29)	Nigeria	West Africa	66	cross-sectional	WHOQOL-BREF	0 to 100	94.03 ± 10.61	77.8
17	Luqman et al. (38)	Nigeria	West Africa	100	Cross-sectional	QOLIE-31	0 to 100	49.69 ± 13.45	88.9
18	Mohamed et al. (42)	Sudan	East Africa	50	Cross-sectional	QOLIE-AD-48	0 to 100	78.95 ± 12.9	88.9
19	Nubukpo et al. (39)	Togo	West Africa	281	Cross-sectional	QOLIE-31	0 to 100	49.5 ± 14.4	100
20	Nubukpo et al. (39)	Benin	West Africa	215	Cross-sectional	QOLIE-31	0 to 100	52.1 ± 33.4	66.7

### Studies characteristics

This systematic review and meta-analysis included 20 studies from SSA, comprising a total of 4,200 participants with sample sizes ranging from 50 to 439. All included studies utilized a cross-sectional study design. Of the 20 studies, eight were conducted in Ethiopia, six in Nigeria, two in Uganda, and one each in Tanzania, Sudan, Togo, and Benin. Geographically, 12 studies were based in East Africa, while eight were from West Africa. In terms of assessment tools, 12 studies used the QOLIE-31 questionnaire (24, 26, 28, 32–39), six used the WHOQOL-BREF scale (21, 27, 29, 40, 41), and two used the HRQoL in Epilepsy for Adolescents (QOLIE-AD)-48 (42, 43). All instruments evaluated HRQoL on a scale of 0 to 100, with higher scores representing better HRQoL (Table 1).

The pooled mean score of health-related quality of life of patients with epilepsy in Sub-Saharan Africa.

**Figure 2:**
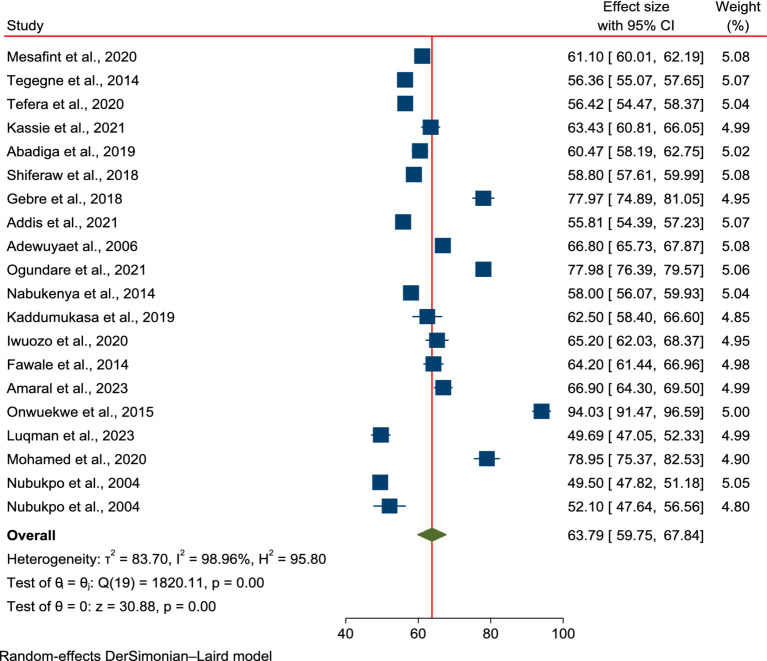
Forest plot of the pooled mean score of HRQoL among patients with epilepsy in SSA.

The pooled mean score of HRQoL among PLWE in SSA countries was 63.79% (95% CI: 59.75–67.84%). Owing to significant heterogeneity across the studies, a random-effects model was applied for the meta-analysis (I^2^ = 98.96%, *p* < 0.001). As shown in Figure 2, 20 studies were included in this meta-analysis to estimate the overall mean score of HRQoL among PLWE in the region.

### Subgroup and meta-regression analysis

The selected studies demonstrated significant heterogeneity (I^2^ = 98.96%, *p* < 0.001), indicating that the variability among the studies exceeded what would be expected. This led to an inconsistent overall estimate of the pooled mean score for HRQoL among PLWE in Sub-Saharan Africa. To address this, a random-effects model was used for the pooled estimates. Meta-regression and subgroup analyses were performed to explore the sources of heterogeneity. Factors such as sample size, study region in Africa, and tools used to assess HRQoL were included in the meta-regression. The sample size was found to be a significant contributor to heterogeneity [SF 3 (heterogeneity).docx].

**Figure 3:**
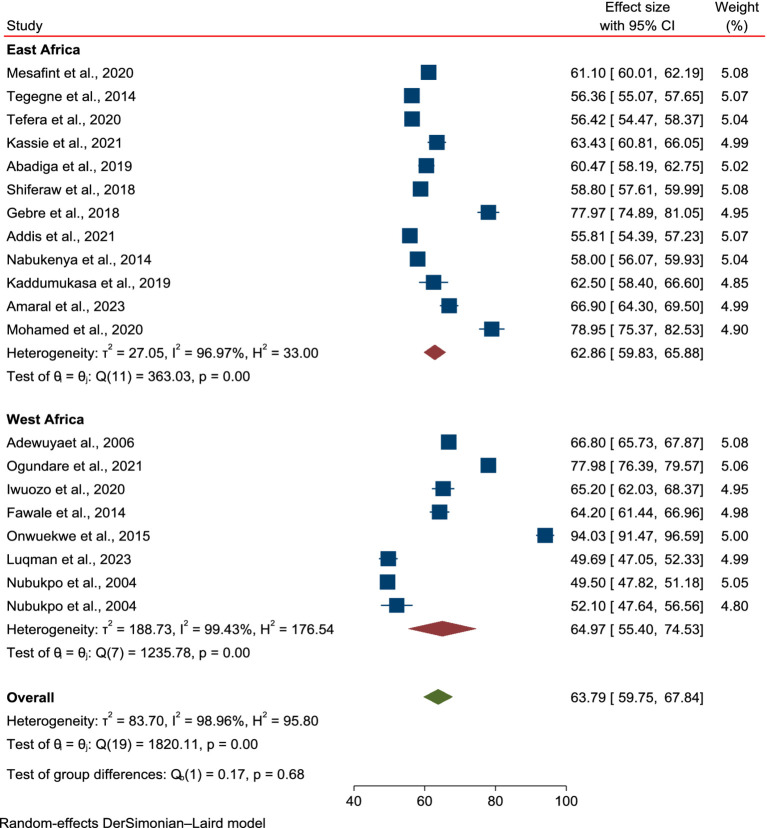
Subgroup analysis based on regions in Africa for the mean score of HRQoL among epilepsy patients in SSA.

**Figure 4:**
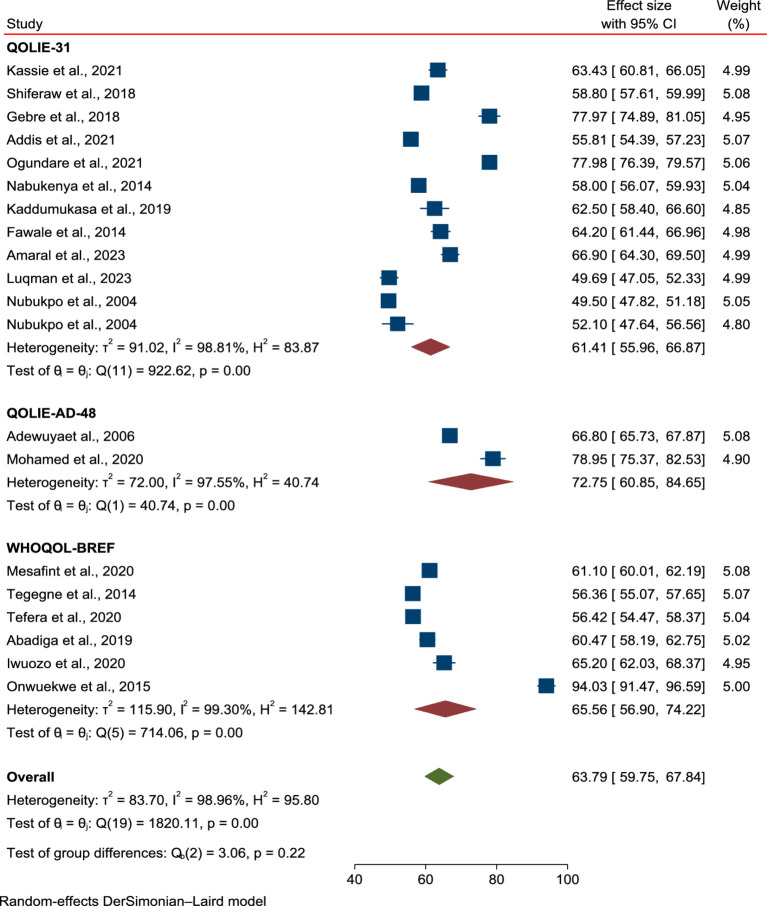
Subgroup analysis based on the tools used to assess HRQoL among epilepsy patients in SSA.

The subgroup analysis based on study regions in Africa and the tools used to assess HRQoL in PLWE revealed no significant differences in the mean scores between East and West African countries. The mean score of HRQoL for patients in East Africa was 62.86 (95% CI: 59.83–65.88, *p* = 0.00), while in West Africa it was 64.97 (95% CI: 59.4–74.55, *p* = 0.00; Figure 3), with no reduction in heterogeneity (I^2^ = 98.96%, *p* < 0.001). Similarly, stratification by the tools used to assess HRQoL showed no changes in mean score or heterogeneity: QULIE-31 yielded 61.41 (95% CI: 55.96–66.87, *p* = 0.00), QOLE-AD-48 gave 72.75 (95% CI: 60.85–84.65, *p* = 0.00), and WHOQOL-BREF produced 65.56 (95% CI: 56.9–74.22, *p* = 0.00), with high heterogeneity (I^2^ = 98.86%, *p* < 0.001; Figure 4).

**Figure 5:**
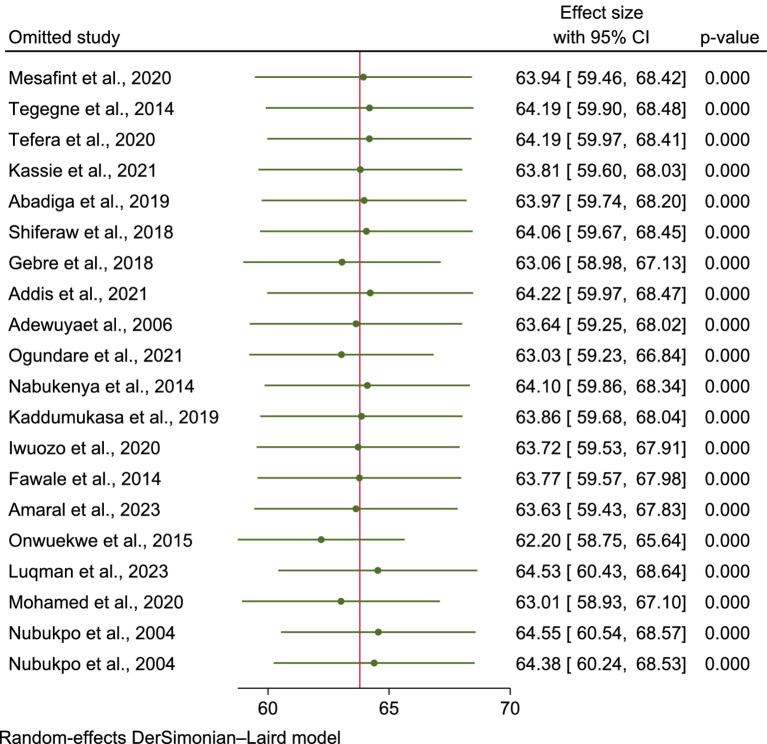
Leave-one-out sensitivity analysis.

A Galbraith plot was generated to identify any studies that deviated significantly from the others, which could contribute to heterogeneity. As shown in Figure 5, 20 studies were analyzed, with one study noticeably displaced in the plot. However, after removing this outlier, the heterogeneity across the studies remained high (I^2^ = 98.51%, *p* = 0.00), indicating no reduction in variability [SF 4 (Galbraith plot).docx]. Sensitivity analysis.

To assess the influence of each study on the pooled mean score of HRQoL among PLWE, a leave-one-out meta-analysis was performed. The analysis showed that when each study was excluded, the pooled mean score remained within the confidence interval of the overall estimate, suggesting that no single study significantly affected the pooled result or the statistical significance (Figure 5).

### Publication bias

Despite the asymmetry of the funnel plot, a formal evaluation using Egger’s test did not indicate significant publication bias in reporting the mean HRQoL score in PLWE. Publication bias was not statistically significant, with a *p*-value of 0.321 (SF 5 (Publication bias).docx).

### Factors associated with poor quality of life patients in people living with epilepsy in SSA

Key findings from this meta-analysis indicated that anxiety (*β* = −4.762, *p* = 0.0029), depression (*β* = −4.591, *p* < 0.0001), and uncontrolled seizures (*β* = −4.321, p < 0.0001) significantly contributed to reduced HRQoL (Table 2). These factors demonstrated not only strong statistical significance but also high levels of heterogeneity, with I^2^ values of 95.91, 95.16, and 75.78%, respectively, indicating variability in the study results. Additionally, perceived stigma negatively impacted HRQoL (*β* = −3.047, *p* = 0.0017), while comorbidity showed a profound effect (*β* = −9.176, *p* < 0.0001), making it the most important factor among those analyzed.

**Table 2:** Factors associated with poor quality of life in patients with epilepsy in Sub-Saharan Africa.

Variables	Model	Beta Coefficient (95% CI)	*p*-value	Heterogeneity (I^2^%)	Number of studies	Egger Test (Publication bias)
No formal education	Random	−2.082 (−9.675, 5.510)	0.5909	88.35	3	0.6228
Anxiety	Random	−4.762 (−7.893, −1.632)	0.0029	95.91	4	0.0000
Depression	Random	−4.591 (−6.948, −2.233)	0.0000	95.16	6	0.0000
Lack of family/social support	Random	−0.344 (−1.088, 0.400)	0.3644	87.87	4	0.0000
Antiepileptic drug side effect	Random	−0.678 (−1.437, 0.081)	0.0802	88.03	5	0.0005
uncontrolled seizure	Random	−4.321 (−5.916, −2.726)	0.0000	75.78	9	0.0038
Perceived stigma	Random	−3.047 (−4.947, −1.147)	0.0017	92.51	5	0.0000
Polypharmacy	Random	−0.915 (−2.752, 0.923)	0.3293	76.86	5	0.1935
Comorbidity	Random	−9.176 (−13.863, −4.489)	0.0000	76.59	3	0.5259
Family history of epilepsy	Random	−5.093 (−8.196, −1.989)	0.0013	0.00	2	0.8548

Conversely, other variables, such as no formal education (*β* = −2.082, *p* = 0.5909), lack of family/social support (*β* = −0.344, *p* = 0.3644), and polypharmacy (*β* = −0.915, *p* = 0.3293), were not statistically significant, suggesting that they may not play a critical role in determining HRQoL for these patients in SSA. The side effects of antiepileptic drugs (*β* = −0.678, *p* = 0.0802) also did not reach significance, although the *p*-value was close to the threshold, indicating potential relevance. Lastly, a family history of epilepsy showed a statistically significant negative impact on HRQoL (*β* = −5.093, *p* = 0.0013).

Assessment of publication bias using Egger’s test revealed a significant bias for anxiety (*p* < 0.0001), depression (*p* < 0.0001), perceived stigma (*p* < 0.0001), and uncontrolled seizures (*p* = 0.0038) suggesting that these factors may be underreported in the literature. Overall, anxiety, depression, uncontrolled seizures, comorbidities, and perceived stigma emerged as critical factors significantly affecting HRQoL for PLWE in SSA, highlighting the need for comprehensive management strategies that address these psychological and social dimensions of care.

## Discussion

This systematic review and meta-analysis aimed to determine the pooled mean score of HRQoL and the factors associated with it in PLWE in SSA. A total of 20 cross-sectional studies involving 4,200 participants with sample sizes ranging from 50 to 439 were retrieved and analyzed from various SSA countries. The findings from the studies examined in this review highlight several key factors that contribute to the decreased HRQoL in PLWE in SSA countries. Uncontrolled seizures were identified in nine studies, depression in six studies, perceived stigma in five studies, anxiety in four studies, and comorbidity in three studies.

The pooled mean score of HRQoL among PLWE in SSA countries was 63.79% (95% CI: 59.75–67.84%). Regarding specific countries, Togo recorded the lowest HRQoL score, measured at 49.5 ± 14.4 (39), suggesting significant challenges faced by individuals in that area regarding their health and well-being. In contrast, Nigeria achieved the highest HRQoL score at 94.03 ± 10.61 (29), indicating that individuals in this country experience a considerably better HRQoL compared to their counterparts in Togo. These variations reflect the diverse circumstances and support systems available to the PLWE in different countries within SSA.

Regarding the subgroup analysis based on study regions in Africa, there were no significant differences in the mean scores between East and West African countries. The mean HRQoL score for patients in East Africa was 62.86 (95% CI: 59.83–65.88, *p* = 0.001), whereas, in West Africa, it was 64.97 (95% CI: 59.4–74.55, *p* = 0.001), with no reduction in heterogeneity (I^2^ = 98.96%, *p* < 0.001). The findings indicate that the mean HRQoL value among PLWE remained unaffected by regional variations in the study conducted across countries in SSA. Moreover, subgroup analysis based on the tools used to assess HRQoL in PLWE showed no changes in the mean score or heterogeneity. QULIE-31 yielded 61.41 (95% CI: 55.96–66.87, *p* = 0.00), QOLE-AD-48 gave 72.75 (95% CI: 60.85–84.65, *p* = 0.001), and WHOQOL-BREF produced 65.56 (95% CI: 56.9–74.22, *p* = 0.001), with heterogeneity remaining high (I^2^ = 98.86%, *p* < 0.001). The review findings indicated that there was no statistically significant difference in HRQoL based on the tool used to assess HRQoL in PLWE. Therefore, we can use one of the validated tools mentioned above to assess HRQoL in SSA countries.

Psychiatric disorders are more prevalent in PLWE than in the general population and among individuals with other neurological diseases (44). In contrast, epilepsy significantly impairs physical health and causes psychological disturbances such as depression and anxiety, which adversely affect patients’ HRQoL (41). This meta-analysis indicated that PLWE who had comorbid anxiety (*β* = −4.762, *p* = 0.0029) experienced poor HRQoL in four studies (22, 24, 40, 45). Symptoms of anxiety disorders have the most significant impact on HRQoL compared with comorbid conditions. Higher levels of anxiety traits are associated with poorer HRQoL in various areas, including emotional and mental well-being, pain, energy and vitality, cognitive function, and social functioning in PLWE (46, 47). In addition, subjective anxiety has a greater effect than short-term seizure control on HRQoL scores of PLWE (48). These factors should be simultaneously considered when evaluating the effects of treatment on HRQoL.

Depression frequently occurs alongside epilepsy and affects the HRQoL of many patients (49). In this meta-analysis, depression (*β* = −4.591, *p* < 0.0001) was associated with poor HRQoL in PLWE in six studies (22, 24, 34, 43, 45, 50). Various depressive disorders can occur in PLWE and are often related to hyperactivity of the hypothalamic–pituitary–adrenal axis, neuroinflammation, and disruptions in serotonin transmission, among other factors (51, 52). Additionally, certain ASDs medications may have adverse psychotropic effects, potentially causing or worsening depression, which can ultimately compromise HRQoL in PLWE (53).

One of the reasons for the poor HRQoL in PLWE is the unpredictable nature of seizures, particularly when they are uncontrolled. Research has shown that uncontrolled seizures are associated with a decline in HRQoL (54). In this meta-analysis, uncontrolled seizures (*β* = −4.321, *p* < 0.0001) significantly contributed to reduced HRQoL in nine studies (22, 24, 28, 32, 34, 36, 40, 45). Fears and worries about experiencing seizures in public create a constant source of anxiety for PLWE (55, 56). They often dread the possibility of being mocked, scarring others, or witnessing others’ helplessness during a seizure. The sudden and unpredictable nature of seizures, along with concerns about potential injuries during an episode, heightens anxiety (57). This fear contributes to a feeling of lack of control over their seizures, which negatively affects their HRQoL (56, 58).

Stigma has debilitating effects on HRQoL in PLWE and the pooled mean score of perceived stigma and self-stigma among PLWE in SSA is 43.9%, it is linked to various challenges in their daily lives, such as internalizing negative attitudes, exhibiting decreased adherence to treatment, and experiencing higher rates of unemployment, leading to lower self-esteem and levels of hope (59). In many societies in SSA, there is a belief that epilepsy is contagious. This misconception can lead patients to feel the need to isolate themselves due to the stigma associated with their condition, resulting in a loss of confidence and feelings of embarrassment (60). Such stigma creates significant challenges for individuals in accessing education, finding stable employment, and forming intimate relationships (61). In this meta-analysis, perceived stigma negatively impacted HRQoL (*β* = −3.047, *p* = 0.0017) in five studies (22, 24, 36, 40, 45). Distress from stigma often surpasses that of the disease itself, leading to feelings of guilt and increased depression. In PLWE, stigma arises from seizure unpredictability and social exclusion resulting from negative societal attitudes (62). This can result in challenges related to education, starting a family, and finding employment, even when these opportunities are feasible (63). Epilepsy significantly affects HRQoL and overall well-being due to the nature of seizures, which can be unpredictable and may affect awareness. Additionally, PLWE often experiences psychiatric and cognitive challenges. Changes in personal, social, and community aspects of life can further affect a person’s HRQoL, leading to limitations in autonomy and an increased perception of stigma (64).

In this meta-analysis, comorbidity has a significant negative impact on HRQoL in PLWE (*β* = −9.176, *p* < 0.0001) in three studies (21, 32, 40), and demonstrated the most substantial effect among the factors analyzed. This detrimental effect was observed in three different studies. Additionally, a family history of epilepsy also showed a statistically significant negative effect on HRQoL (*β* = −5.093, *p* = 0.0013) in two studies (32, 34).

The selected studies exhibited significant heterogeneity (I^2^ = 98.96%, *p* < 0.001), indicating that the variability among the studies was greater than what would typically be expected. To identify the source of this heterogeneity, the author analyzed factors such as sample size, country, study region in Africa, and the tools used to assess HRQoL using a random effects model. In this meta-analysis, only sample size was found to be a significant source of heterogeneity (*p* = 0.048). A leave-one-out meta-analysis was conducted to evaluate the impact of each study on the pooled mean HRQoL score in PLWE. This analysis indicated that no single study significantly affected the overall results. Furthermore, an assessment of publication bias showed no statistically significant bias (*p* = 0.321). Although there was some asymmetry in the funnel plot, a formal evaluation using Egger’s test did not indicate significant publication bias in the reporting of mean HRQoL scores for PLWE.

Challenges in improving the HRQoL of PLWE in SSA stem from a widespread belief that epilepsy is caused by demonic possession (65). This belief contributes to a significant social stigma surrounding the condition. In many societies in SSA, there is a belief that epilepsy is contagious (60). Such perceptions can fuel fear and misunderstanding within communities, often resulting in the isolation of those with epilepsy. Additionally, traditional healing practices may be sought instead of, or alongside, medical treatment, highlighting the cultural nuances that affect how epilepsy is perceived and managed in this region. One of the main challenges in SSA countries is that a significant portion of the population resides in rural areas. In these regions, the impact of inadequate healthcare on the HRQoL for PLWE is notably more pronounced. Additionally, resources are limited, and access to healthcare services is insufficient, which greatly affects the HRQoL of PLWE (5).

The potential interventions to enhance HRQoL among PLWE involve targeting psychological approaches, including cognitive-behavioral and behaviorally based interventions and mindfulness-based interventions (such as acceptance and commitment therapy) (65, 66). The other potential intervention strategy will be (psycho-) educational interventions that will increase knowledge about epilepsy, its comorbidities, and its treatments, as well as the functions and activities of the brain (67–69). The HRQoL for PLWE in low and middle-income countries is significantly correlated with levels of resilience and internalized stigma. Enhancing resilience may effectively mitigate the adverse effects of perceived stigma on HRQoL, thereby contributing to improved overall well-being for PLWE in these regions (70, 71).

## Limitations of the study

This systematic review has certain limitations. It only included papers published in English, which restricted the scope owing to the relevance of the topic and most studies utilized a variety of instruments to measure HRQoL, which compromises the conclusions. Additionally, the number of studies from Ethiopia is relatively high, and the review focused solely on cross-sectional studies, which may have affected the generalizability of the findings.

## Conclusion

This review found a moderate pooled mean score of HRQoL among PLWE in SSA countries. Factors that negatively affect HRQoL in these regions include anxiety, depression, uncontrolled seizures, comorbidities, and a family history of epilepsy. This study may provide valuable insights to relevant organizations focused on the early screening and management of HRQoL in PLWE. Additionally, it is important to include screening and treatment of anxiety and depression as part of regular epilepsy care management. Our study highlights the need for further studies, particularly those using longitudinal designs.

## Data Availability

The original contributions presented in the study are included in the article/Supplementary material, further inquiries can be directed to the corresponding author.
