# Supplementary material for: Health-related quality of life and associated factors among epilepsy patients in sub-Saharan Africa: a systematic review and meta-analysis
Source: Front Neurol. 2025 Mar 5;16:1546911. doi: 10.3389/fneur.2025.1546911 (PMC11921783; doi:10.3389/fneur.2025.1546911)
Supplement: Supplementary file 1 [file Table_1.docx]

Additional file 1: Risk of bias within studies using the Joanna Briggs Institute criteria’s

| Included Studies | JBI quality assessment criteria’s | | | | | | | | | Total Score % |
| --- | --- | --- | --- | --- | --- | --- | --- | --- | --- | --- |
|  | Q1 | Q2 | Q3 | Q4 | Q5 | Q6 | Q7 | Q8 | Q9 |  |
| Mesafint et al., 2020 | Y | Y | Y | Y | Y | Y | Y | Y | Y | 100 |
| Tegegne et al., 2014 | Y | Y | N | Y | Y | Y | Y | Y | Y | 88.9 |
| Tefera et al., 2020 | Y | Y | Y | N | Y | Y | Y | N | Y | 77.7 |
| Kassie et al., 2021 | Y | Y | Y | Y | Y | Y | Y | Y | Y | 100 |
| Abadiga et al., 2019 | Y | Y | Y | Y | Y | Y | U | Y | Y | 88.9 |
| Shiferaw et al., 2018 | Y | Y | N | Y | Y | U | Y | Y | Y | 77.7 |
| Gebre et al., 2018 | Y | N | Y | Y | Y | Y | Y | Y | Y | 88.9 |
| Addis et al., 2021 | Y | U | N | Y | Y | Y | Y | Y | Y | 77.8 |
| Adewuyaet al., 2006 | Y | U | Y | Y | Y | Y | Y | U | Y | 77.7 |
| Ogundare et al., 2021 | Y | Y | Y | Y | Y | Y | N | Y | Y | 88.9 |
| Nabukenya et al., 2014 | Y | Y | N | Y | Y | Y | U | Y | Y | 77.7 |
| Kaddumukasa et al., 2019 | Y | Y | N | Y | Y | Y | Y | Y | Y | 88.9 |
| Iwuozo et al., 2020 | Y | U | N | Y | Y | Y | Y | Y | Y | 77.7 |
| Fawale et al., 2014 | Y | Y | Y | Y | Y | Y | U | Y | Y | 88.9 |
| Amaral et al., 2023 | Y | Y | Y | Y | Y | Y | U | Y | Y | 88.9 |
| Onwuekwe et al., 2015 | Y | Y | Y | U | Y | Y | N | Y | Y | 77.8 |
| Luqman et al., 2023 | Y | Y | Y | Y | Y | Y | U | Y | Y | 88.9 |
| Mohamed et al., 2020 | Y | Y | N | Y | Y | Y | U | Y | Y | 88.9 |
| Nubukpo et al., 2004 | Y | Y | Y | Y | Y | Y | Y | Y | Y | 100 |
| Nubukpo et al., 2004 | Y | Y | Y | Y | Y | Y | Y | Y | Y | 100 |
| Note: Y - Yes, N - No, U – Unclear, NA-Not applicable  Q1= Was the sample frame appropriate to address the target population?  Q2= Were study participants sampled in an appropriate way?  Q3= Was the sample size adequate?  Q4= Were the study subjects and the setting described in detail?  Q5= Was the data analysis conducted with sufficient coverage of the identified sample?  Q6= Were valid methods used for the identification of the condition?  Q7= Was the condition measured in a standard, reliable way for all participants?  Q8= Was there appropriate statistical analysis?  Q9= Was the response rate adequate, and if not, was the low response rate managed appropriately? | | | | | | | | | | |
