# Supplementary material for: Health-related quality of life and associated factors among epilepsy patients in sub-Saharan Africa: a systematic review and meta-analysis
Source: Front Neurol. 2025 Mar 5;16:1546911. doi: 10.3389/fneur.2025.1546911 (PMC11921783; doi:10.3389/fneur.2025.1546911)
Supplement: Supplementary file 3 [file Table_3.docx]

Meta-regression analysis of the studies based on sample size, country, study regions in Africa, and the tools used to assess HRQoL

| Variables | Coefficient | Std. err. | z | P>\|z\| | [95% conf. interval] |
| --- | --- | --- | --- | --- | --- |
| Sample size | -0.0386389 | 0.0195289 | -1.98 | 0.048 | -0.0769147 -0.000363 |
| Country | -1.027052 | 1.519556 | -0.68 | 0.499 | -4.005328 1.951223 |
| Study region in Africa | -1.595072 | 4.841752 | -0.33 | 0.742 | -11.08473 7.894587 |
| Tools | 2.367102 | 2.535678 | 0.93 | 0.351 | -2.602736 7.33694 |
| _Cons | 73.42956 | 13.74579 | 5.34 | 0.000 | 46.48831 100.3708 |

Test of residual homogeneity: Q_res = chi2(15) = 1374.23 Prob > Q_res = 0.0000.
