# Supplementary material for: Health-related quality of life and associated factors among epilepsy patients in sub-Saharan Africa: a systematic review and meta-analysis
Source: Front Neurol. 2025 Mar 5;16:1546911. doi: 10.3389/fneur.2025.1546911 (PMC11921783; doi:10.3389/fneur.2025.1546911)
Supplement: Supplementary file 4 [file Table_4.docx]

Galbraith plot of random effect modal
